# FT3 and FT3/FT4 ratio are decreased and not compensated by levothyroxine treatment in TKI-treated thyroid cancer patients

**DOI:** 10.1530/ERC-24-0323

**Published:** 2025-05-14

**Authors:** Tommaso Porcelli, Cristina Luongo, Anna Cerbone, Carmine Di Luccio, Maria Angela De Stefano, Domenico Salvatore

**Affiliations:** ^1^ Department of Public Health, University of Naples “Federico II”, Naples, Italy; ^2^ Department of Clinical Medicine and Surgery, University of Naples “Federico II”, Naples, Italy

**Keywords:** tyrosine kinase inhibitors, hypothyroidism, thyroid hormone treatment, LT4 + LT3 combination, deiodinases

## Abstract

Tyrosine kinase inhibitors (TKIs) can alter thyroid hormone levels in multiple cancer contexts. The aim of this study was to analyse the efficacy of levothyroxine (LT4) treatment to normalise thyroid hormone levels in thyroid cancer patients with increased thyroid-stimulating hormone (TSH) after TKI initiation. Forty-five consecutive patients were enrolled. One month after TKI initiation, TSH increased from 0.21 (IQR: 0.07–0.8) to 1.09 mIU/L (IQR: 0.16–4.1) (*P* < 0.0001), and the FT3/FT4 ratio decreased from 2.1 (IQR: 1.86–2.66) to 1.92 (IQR 1.69–2.3) (*P* < 0.001) compared to baseline. During the first year of TKI treatment, in 23 patients (51%), TSH increased >4 mIU/L with a concomitant decrease in both FT3 and FT4. An increase in the LT4 daily dose of 0.62 ± 0.44 μg/kg normalised TSH to baseline levels in all patients, but in 11 patients FT3 remained lower compared to baseline, i.e. 2.53 (IQR: 2.4–2.75) versus 3.5 pg/mL (IQR: 3.18–3.84), respectively (*P* = 0.001). Following this observation, in three consecutive patients with increased TSH and reduced FT3 during TKI treatment, we changed the LT4 monotherapy into an LT4 + LT3 combination at a ratio of 11:1. Two months later, serum TSH, FT3 and FT4 were all similar to baseline (*P* > 0.05 in all determinations). In conclusion, TKI-related increase in TSH was associated with decreased FT3 and FT3/FT4 ratio. This was not compensated by an increase in LT4 in half of the cases. The clinical impact of compensating for the low T3 levels in these patients should be addressed in prospective trials.

## Introduction

Tyrosine kinase inhibitors (TKIs) are anti-cancer drugs used in numerous tumour settings. They share a common mechanism of action that consists in binding to kinases involved in tumourigenesis in order to inhibit their activity (Attwood *et al.* 2021). As a class adverse event, TKIs present an increase in circulating levels of thyroid-stimulating hormone (TSH), which may evolve into overt hypothyroidism (Lechner *et al.* 2018). This adverse event was found with the first generation of multikinase angiogenesis inhibitors, but it also occurs with more recent highly selective inhibitors such as the RET-inhibitor selpercatinib (Wirth *et al.* 2020).

The mechanisms by which TKIs increase serum TSH level have not been fully elucidated. We previously demonstrated in preclinical models that this may be at least partially due to the inhibition of type 2 deiodinase (D2), an enzyme which activates the prohormone T4 into its active form T3 (Porcelli *et al.* 2023). Some studies have shown a TKI-dependent increase in the activity of type 3 deiodinase (D3), which inactivates T4 into reverse T3 (Abdulrahman *et al.* 2010). Other studies have shown a direct damage to the thyroid gland (Desai *et al.* 2006), but TKI-related hypothyroidism occurs both in patients with a normal thyroid gland and in thyroidectomised patients who are on therapy with levothyroxine (LT4) (Makita & Iiri 2013).

Thyroidectomised patients treated with LT4 are indeed more susceptible to interference on the thyroid axis since they are entirely dependent on peripheral deiodinases for the activation of LT4 into T3 (De Stefano *et al.* 2023). In this context, a marked reduction in circulating T3 levels was observed in LT4-treated thyroid cancer patients during treatment with selpercatinib, supporting the concept that the inhibition of D2 activity might be a relevant pathogenetic mechanism for the TKI-related increase in serum TSH level in these patients (Boucai *et al.* 2022). Maintaining adequate T3 levels in thyroidectomised patients might be crucial, as some tissues (e.g. liver) do not express D2 and rely only on plasma for their cellular T3 supply (Salvatore *et al.* 2022). In T3-dependent tissues, a reduction of about 20% of circulating T3 seems to be already clinically relevant, as reported in hypothyroid LT4-treated patients with normal TSH but low T3, who had increased hypothyroid symptoms and worse metabolic parameters compared to controls with normal T3 (Peterson *et al.* 2016, Ito *et al.* 2019). In the context of TKI-treated patients, this could potentially worsen the tolerability of the treatment and the patient’s quality of life.

It is unclear whether all TKIs share a common pattern in altering the serum thyroid hormone profile. Importantly, assuming that the alterations which occur in LT4-treated patients during a TKI treatment are mostly due to defects in LT4 deiodination, there is a lack of knowledge on which thyroid hormone treatment is the most appropriate. Here, we investigated the changes in circulating T3 and T4 levels in patients with a TKI-related increase in TSH level, and their modifications following an increasing dose of LT4. To this aim, we retrospectively analysed circulating thyroid hormone levels in a series of 45 consecutive thyroidectomised patients treated with various TKIs for metastatic and progressive thyroid cancer. In addition, we explored the possibility of using a combination therapy of LT4 + liothyronine (LT3) instead of increasing the dose of LT4 in three patients with increased TSH during TKI treatment.

## Methods

### Patients and data collection

We examined the clinical charts of 89 patients referred from January 2016 to October 2024 to Federico II University Hospital for a metastatic thyroid cancer. We focused on 45 patients who started a first-line treatment with TKIs and were then followed at our institution, and extracted demographic and clinical data (gender; age at cancer diagnosis; age, weight and ECOG performance status at TKI initiation and during the first year of TKI treatment; date of death or date of last follow-up visit), pathological information (histotype; primary tumour size; synchronous or metachronous distant metastases; distant metastatic sites; and number of metastatic sites per patient at TKI initiation), serum thyroid hormone levels (at baseline before TKI initiation and all measurements during the first year of TKI treatment), and treatment details (type and initial dose of TKI; thyroid hormone treatment dose before TKI initiation and during TKI treatment).

These 45 patients formed the basis of this report. All patients were followed up every 1–3 months after TKI initiation with clinical examinations, serum thyroid hormone level determinations in our laboratory and standardised imaging techniques to evaluate the tumour volume over time.

This study was conducted in accordance with the Declaration of Helsinki and was approved by the ethical committee of Federico II University Hospital, ‘Comitato Etico Campania 3’ (protocol number 152/2023).

### Treatment with TKIs

The decision to initiate a treatment with TKIs was based on the presence of clinically relevant disease progression and/or disease-related symptoms. The disease progression according to RECIST v1.1 was assessed by comparing imaging modalities performed at an interval of 3–12 months depending on tumour growth rate (Eisenhauer *et al.* 2009). The choice of the TKI and the TKI starting dose was based for each patient on the tumour histology and the presence of an actionable mutation, the general clinical conditions, ECOG performance status and comorbidities (Porcelli *et al.* 2025).

### Serum thyroid hormones measurement

In all patients, baseline serum thyroid hormone levels were measured within 1 month before the initiation of TKI treatment and every 1–3 months thereafter. Patients were instructed not to take thyroid hormone therapy on the morning of sampling for serum thyroid hormone analysis. Serum thyroid hormones (TSH, total T3 and total T4, free T3 (FT3) and free T4 (FT4)) were analysed using the Atellica IM analysers (Siemens Healthcare Diagnostics Inc, USA), which are competitive immunoassay tests based on direct chemiluminescent technology. The reference intervals, the measuring intervals and the coefficients of variation for these tests are listed in Supplementary Table 1 (see section on Supplementary materials given at the end of the article).

### Levothyroxine therapy and definition of TSH increase

Levothyroxine (LT4) was administered in all patients with follicular-cell derived thyroid cancer (*n* = 40) to achieve mild-to-moderate or complete TSH suppression (Haugen *et al.* 2016). In patients with metastatic medullary thyroid cancer (*n* = 5), LT4 was administered to maintain TSH levels within the reference range (Wells *et al.* 2015). All patients were instructed to take the LT4 therapy in the morning, on an empty stomach and at least 30 min before breakfast. LT3 was administered twice daily, the first fraction together with the LT4 in the morning and the second fraction 1 h before dinner. According to the Common Terminology Criteria for Adverse Events v.5, we considered an increase in TSH to a value >4 mIU/L as biological hypothyroidism. In patients with follicular-cell derived thyroid cancer, the daily dose of LT4 was increased by 20% after TKI initiation if serum TSH exceeded 0.5 mIU/L. In patients with a serum TSH level <0.5 mIU/L, the daily dose of LT4 was left unchanged and the thyroid hormone levels were controlled 1–3 months later. Based on the coefficients of variation reported with the Atellica IM assay, we set a threshold of at least ±0.5 pg/mL to consider a change in individual FT3 values as clinically significant after the initiation of TKI treatment compared to baseline.

### Statistical analysis

All variables were analysed with the Kolmogorov–Smirnov test to assess normality before data analysis. Median and interquartile range or mean ± standard deviation was used for non-normally distributed data, as appropriate. Continuous variables, including age, primary tumour size and LT4 dose at TKI initiation, were analysed by Mann–Whitney U test. Categorical variables were compared using the Fisher Exact Probability. Differences in serum thyroid hormone values before and after any change in treatment were analysed with the Wilcoxon matched-pairs signed rank test. A *P*-value of <0.05 was considered statistically significant. The statistical analyses were conducted using the GraphPad Prism version 10.1 software.

## Results

### Patient and treatment characteristics

Forty-five consecutive patients who received a TKI treatment for progressive and/or symptomatic metastatic thyroid cancer were included in this study. Patient characteristics are listed in Table 1. Thirty-two patients (71%) were treated with lenvatinib, in 21 cases at initial doses of 24 mg and in 11 cases at reduced initial doses of ≤14 mg because of lesions at risk in seven cases, ECOG ≥2 in two cases and advanced age (>85 years) in two cases. Six patients (13%) received the combination of dabrafenib (320 mg/day) + trametinib (2 mg/day), in all cases at the full doses. Five patients (11%) received selpercatinib, four cases at 320 mg daily and one case at 160 mg daily because of atrial fibrillation and concomitant treatment with amiodarone. Finally, two patients (4%) received vandetanib, both starting at 300 mg daily. Thirty-nine patients (87%) were treated with a TKI for at least 1 consecutive year, while 6 patients (13%) discontinued TKI before 1 year, after a median time of 9 months (6.5–11), due to adverse event (*n* = 1), disease progression (*n* = 1), death from disease (*n* = 3), and death unrelated to the disease (*n* = 1).

**Table 1:** Characteristics of the study patients.

	All (*n* = 45)	Increased TSH <4 mIU/L (*n* = 22)	Increased TSH >4 mIU/L (*n* = 23)	*P* value
Gender (female; %)	18 (40%)	9 (41%)	10 (43%)	0.95
Age at cancer diagnosis (years)	59 (49–65)	58.5 (48–66)	61 (50–65)	0.87
Histotype				
Follicular-cell derived thyroid cancer	40 (89%)	21 (95%)	19 (83%)	0.17
Medullary thyroid cancer	5 (11%)	1 (5%)	4 (17%)	
Primary tumour size (cm)	4 (2.4–5.8)	3.1 (2.2–4.6)	5 (3–7)	**0.05**
Synchronous metastases (yes)	16 (36%)	4 (18%)	12 (54%)	**0.04**
Metastatic sites per patient at TKI initiation (mean)	2.2 ± 0.9	2.3 ± 0.98	2.1 ± 0.85	0.45
Metastatic sites at TKI initiation				
Mediastinal lymph nodes	26 (58%)	14 (64%)	12 (52%)	0.23
Lung	36 (80%)	18 (82%)	18 (78%)	0.30
Bone	17 (38%)	7 (32%)	10 (43%)	0.57
Other	8 (18%)	3 (14%)	5 (22%)	0.57
Age at TKI initiation (years)	67 (58–74)	69.5 (60–75)	64 (58–72)	0.35
ECOG at TKI initiation				
0–1	41 (91%)	21 (95%)	20 (87%)	0.32
≥2	4 (8%)	1 (5%)	3 (13%)	
Levothyroxine dose at TKI initiation (μg/kg)	1.81 ± 0.37	1.82 ± 0.38	1.76 ± 0.34	0.84
TKI type				
Lenvatinib	32 (71%)	16	16	0.82
Dabrafenib + trametinib	6 (13%)	5	1	0.07
Selpercatinib	5 (11%)	1	4	0.17
Vandetanib	2 (4%)	0	2	0.16

Bold indicates statistical significance

### Baseline thyroid hormone levels

Patients with follicular-cell derived thyroid carcinoma had baseline TSH values <0.1 mIU/L in 16 cases, between 0.1 and 0.5 mIU/L in 13 cases and between 0.5–2.2 mIU/L in 11 cases. The differences in mean FT3/FT4 ratio according to serum TSH level were not statistically significant (*P* > 0.05 in all determinations): it was 2.18 ± 0.46 in patients with TSH <0.1 mIU/L, 2.26 ± 0.65 in those with TSH between 0.1–0.5 mIU/L and 2.43 ± 0.52 in those with TSH >0.5 mIU/L. All patients with medullary thyroid cancer (*n* = 5) had a TSH value within normal limits (mean value 1.76 ± 1.25 mIU/L, range 0.21–3.4) and had a mean FT3/FT4 ratio of 2.26 ± 0.62.

### Thyroid hormone level alterations after TKI initiation

**Figure 1:**
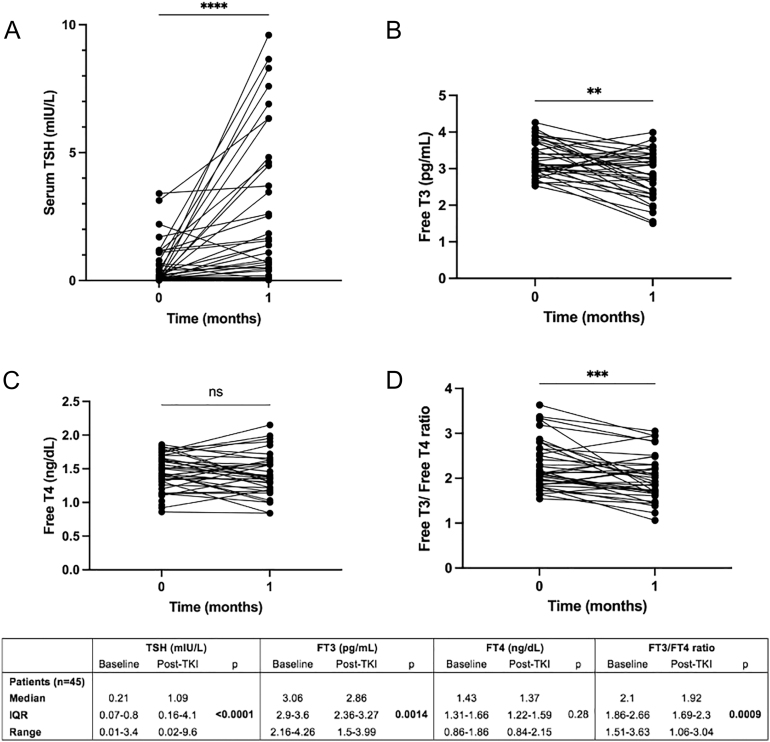
Serum thyroid hormone levels in 45 patients treated with TKIs for an advanced thyroid cancer at baseline and 1 month after treatment initiation.

One month after TKI initiation (median time 33 days; 28–45), all patients showed significant alterations in serum thyroid hormone levels when compared to baseline (Fig. 1). Specifically, median serum TSH level increased from 0.21 (IQR: 0.07–0.8) to 1.09 mIU/L (IQR: 0.16–4.1) (*P* < 0.0001), median FT3 level decreased from 3.06 (IQR: 2.9–3.6) to 2.86 pg/mL (IQR: 2.36–3.27) (*P* < 0.0001), median FT4 level remained similar to baseline, i.e. 1.43 (IQR: 1.31–1.66) versus 1.37 ng/dL (IQR: 1.22–1.59) (*P* = 0.37), and median FT3/FT4 ratio decreased from 2.1 (IQR: 1.86–2.66) to 1.92 (IQR: 1.69–2.3) (*P* < 0.001) (Fig. 1).

**Figure 2:**
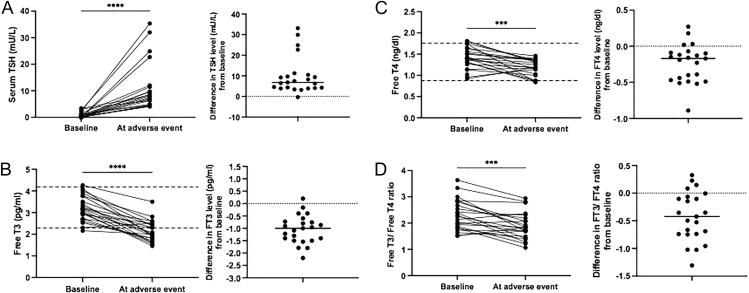
Serum thyroid hormone levels and FT3/FT4 ratio variations compared to baseline in 23 patients who had an increase in serum TSH levels to >4 mIU/L during the first year of treatment with TKIs.

During the first year of treatment with TKIs, 23 of the 45 patients (51%) showed an increase in TSH above the upper limit of the normal range (>4 mIU/L) that required an increase in the daily dose of LT4 treatment (Fig. 2). We did not observe significant differences in the relative percentage of patients who had an increase in TSH level among the three groups of patients with baseline TSH <0.1 mIU/L (44%), between 0.1–0.5 mIU/L (50%) and >0.5 mIU/L (55%) (*P* > 0.05 in all determinations). The median value of TSH at maximal increase was 8.15 mIU/L (IQR: 4.97–9.75), and the median time from TKI initiation to maximal TSH increase was 63 days (28–109) (Table 3). At the time of maximal TSH increase, we observed significant changes in both FT3 and FT4 levels, which showed a median reduction compared to baseline of −1.0 pg/mL (IQR: −1.48 to −0.74) and −0.17 ng/dL (IQR: −0.43 to −0.09), respectively. According to the TKI used for treatment, these alterations in thyroid hormone levels occurred in 16 of the 32 patients (50%) treated with lenvatinib, four of the five patients (80%) treated with selpercatinib, one of the six patients (17%) treated with dabrafenib + trametinib and in both patients treated with vandetanib. We have considered the potential impact of diarrhoea (a frequent adverse event in patients treated with TKI) as a potential mechanism of intestinal malabsorption for low FT4 levels and high TSH levels. Seven of the 16 lenvatinib-treated patients had diarrhoea during the treatment (grade ≤2 in six cases, grade 3 in one case), but only in two of these cases did diarrhoea (grade ≤2 in both cases) precede the appearance of the TSH increase. Moreover, none of the patients who received vandetanib, selpercatinib and dabrafenib + trametinib had diarrhoea. These data suggest that diarrhoea is unlikely to have been responsible for an intestinal malabsorption of LT4.

The remaining 22 patients had TSH levels within the normal range (≤4 mIU/L) during the entire first year of TKI treatment. This group showed slightly but significantly increased median TSH levels 1 month after TKI initiation when compared to baseline, i.e. 0.48 mIU/L (IQR: 0.11–1.45) versus 0.19 mIU/L (IQR: 0.07–0.55) (*P* = 0.01), but no significant variation in FT3/FT4 ratio nor in median FT3 and FT4 levels, i.e. −0.2 pg/mL (IQR: −0.5 to 0.16) and 0.07 ng/dL (IQR: −0.05 to 0.22), respectively (Table 2). In lenvatinib-treated patients – for whom there was a sufficiently large sample of patients – we found no difference in gender, age and ECOG at TKI initiation, and TKI starting dose between patients with TSH increased below 4 mIU/L versus those with TSH increased above 4 mIU/L after TKI initiation (*P* > 0.05 in all comparisons).

**Table 2:** Serum thyroid hormone levels at baseline and after initiation of treatment with tyrosine kinase inhibitors. The patients were classified according to the highest TSH value reached during the first year of treatment.

	TSH (mIU/L)			FT3 (pg/mL)			FT4 (ng/dL)			FT3/FT4 ratio		
	Baseline	Post-TKI	*P*	Baseline	Post-TKI	*P*	Baseline	Post-TKI	*P*	Baseline	Post-TKI	*P*
**TSH increased >4 mIU/L (** ***n*** ** = 23)**												
Median	0.28	8.15		3.16	2.0		1.4	1.19		2.25	1.84	
IQR	0.08–1.27	4.97–9.75	**<0.0001**	2.8–3.64	1.8–2.48	**<0.0001**	1.29–1.6	1.06–1.34	**0.0005**	1.84–2.74	1.54–2.07	**0.0003**
Range	0.001–3.4	4.1–35.33		2.16–4.26	1.46–3.5		0.92–1.81	0.84–1.46		1.51–3.63	1.06–2.94	
**TSH increased ≤4 mIU/L (** ***n*** ** = 22)**												
Median	0.19	0.48		3.03	3.19		1.49	1.53		2.07	2.01	
IQR	0.07–0.55	0.11–1.45	**0.014**	2.92–3.58	2.72–3.4	0.15	1.36–1.7	1.36–1.65	0.38	1.97–2.5	1.72–2.48	0.11
Range	0.03–1.7	0.04–3.46		2.64–3.9	2.2–3.99		0.66–1.86	0.84–1.95		1.65–4.39	1.33–4.05	

Bold indicates statistical significance

### Response to increased dose of levothyroxine in patients with TSH increased after TKI initiation

**Figure 3:**
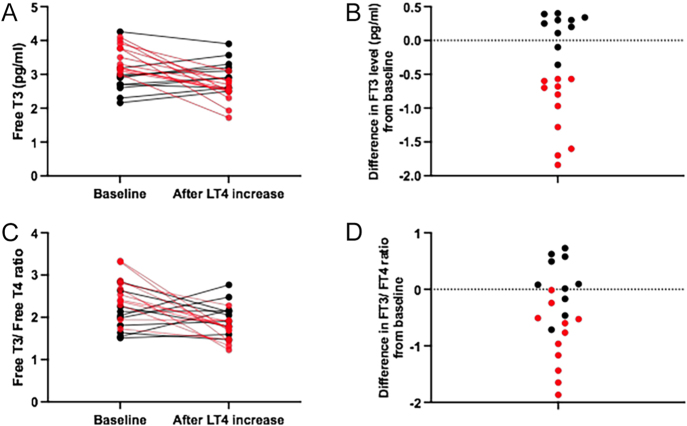
Serum FT3 levels (A and B) and FT3/FT4 ratio (C and D) at baseline and after normalisation of serum TSH by an increased dose of LT4. In 10 (48%) patients, the increase in LT4 dose restored FT3 levels to those before the initiation of TKI treatment (black dots). In 11 (52%) patients, a reduction in FT3 >0.5 pg/mL persisted despite the increase in LT4 dose and the normalisation of TSH levels (red dots).

The 23 patients who showed an increase in TSH levels >4 mIU/L in the first year after TKI initiation required a mean daily increase in LT4 therapy of 0.62 ± 0.44 μg/kg (range: 0.24–1.91; *P* < 0.0001) to restore TSH to baseline levels. Thyroid hormone levels after TSH normalisation were compared to their respective baseline values in the 21 patients for whom these measurements were available (Table 3). Despite similar TSH and FT4 levels compared to baseline in all patients, FT3 levels remained low in 11 patients (52%) (i.e. with a reduction ≥0.5 pg/mL compared to baseline, as stated in ‘Methods’) and were similar to baseline in only ten patients (48%) (Fig. 3). No differences in gender, age, ECOG and LT4 dose at TKI initiation were found between the two groups (*P* > 0.05 in all comparisons).

**Table 3:** Serum thyroid hormone levels at baseline and after normalisation of serum TSH by an increase of LT4 treatment dose.

	TSH (mIU/L)			FT3 (pg/mL)			FT4 (ng/dL)			FT3/FT4 ratio		
	Baseline	After LT4 increase	*P*	Baseline	After LT4 increase	*P*	Baseline	After LT4 increase	*P*	Baseline	After LT4 increase	*P*
**TSH increased >4 mIU/L (** ***n*** ** = 21)**												
Median	0.28	0.46		3.16	2.7		1.4	1.47		2.25	1.79	
IQR	0.08–1.27	0.23–1.1	0.52	2.8–3.64	2.5–2.95	**0.02**	1.29–1.6	1.36–1.67	0.11	1.84–2.74	1.57–2.07	**0.01**
Range	0.001–3.4	0.01–4.3		2.16–4.26	1.72–4.0		0.92–1.8	1.16–1.9		1.51–3.63	1.23–2.77	
**Normalised FT3 (** ***n*** ** = 10)**												
Median	0.93	0.32		2.8	3		1.46	1.59		2.01	2.09	
IQR	0.15–2.5	0.1–0.83	0.99	2.63–2.98	2.68–3.23	0.06	1.09–1.61	1.32–1.76	0.37	1.68–2.22	1.82–2.16	0.57
Range	0.03–3.4	0.01–4.3		2.16–4.26	2.5–3.9		0.92–1.75	1.17–1.9		1.51–2.84	1.47–2.77	
**Reduced FT3 (** ***n*** ** = 11)**												
Median	0.16	0.87		3.5	2.53		1.39	1.46		2.54	1.7	
IQR	0.04–0.94	0.4–1.15	0.2	3.18–3.84	2.4–2.75	**0.001**	1.29–1.47	1.36–1.59	0.24	2.34–2.84	1.46–1.84	**0.001**
Range	0.001–1.19	0.2–2.1		3.0–4.1	1.72–3.1		1.13–1.8	1.16–1.9		1.72–3.34	1.23–2.28	

Bold indicates statistical significance

Among the 22 patients with an increase in TSH levels ≤4 mIU/L in the first year after TKI initiation, a mean daily increase of 0.43 ± 27 μg/kg (range: 0.2–0.8) in LT4 therapy was required in six patients in whom median TSH levels rose from 0.21 mIU/L (IQR: 0.12–0.3) to 1.61 mIU/L (IQR: 0.88–2.4) at first control after TKI initiation, and in two patients where TSH increased below 4 mIU/L after the first month of treatment. In these patients, median FT3 levels after TSH normalisation were similar to those at baseline (*P* > 0.05), with a median variation in FT3 and FT3/FT4 ratio at TSH normalisation of 0.14 pg/mL (IQR: −0.02 to 0.25) and −0.25 (IQR: −0.5 to 0.05), respectively.

### Response to levothyroxine + liothyronine combination in patients with increased TSH after TKI initiation

In the patients showing a reduction in FT3, we hypothesised that a defect in T4-to-T3 conversion occurred as a mechanism contributing to the persistent low serum FT3 concentrations (Boucai *et al.* 2022, Porcelli *et al.* 2023). Therefore, in three consecutive patients with increased TSH levels and decreased FT3 level >0.5 pg/mL after TKI initiation (which were not included in the general analysis); we decided to switch the LT4 monotherapy to an LT4 + LT3 combination. These three patients were submitted to treatment with selpercatinib 320 mg daily. In a mean time of 34 days (range: 9–48) after treatment initiation, all three patients showed an increase in mean TSH levels (2.3 ± 2.1 mIU/L versus 0.48 ± 0.63 mIU/L, respectively), a reduction in mean FT3 levels (2.4 ± 0.2 pg/mL versus 3.6 ± 0.52 pg/mL, respectively), and similar mean FT4 levels compared to baseline (1.35 ± 0.07 versus 1.37 ± 0.1, respectively), resulting in a decreased mean FT3/FT4 ratio (1.85 ± 0.35 versus 2.63 ± 0.47, respectively) (Table 4). Instead of increasing the daily LT4 dose, we reduced the LT4 daily dose by a mean of 0.32 ± 0.22 μg/kg/day and administered LT3 at a mean LT4:LT3 ratio of 11:1 (equivalent to a mean daily dose of LT3 = 0.11 ± 0.02 μg/kg), maintaining the same daily amount of thyroid hormone therapy. Fifty days (range: 23–64) after the switch to LT4 + LT3 combination treatment, TSH and FT4 levels returned to levels similar to those at baseline, as well as FT3 levels and FT3/FT4 ratio (2.47 ± 0.15 versus 2.63 ± 0.47 at baseline) (Table 4). No adverse events related to LT3 supplementation were observed.

**Table 4:** Serum thyroid hormone levels and thyroid hormone replacement therapy in three patients submitted to treatment with selpercatinib at 320 mg daily. Values outside the normal range are shown in bold.

**Patient 1**	**Baseline**	**After selpercatinib start**	
Timepoint		0.3 months	1.9 months
Daily dose of LT4 (μg/kg)	1.92	1.92	1.35
Daily dose of LT3 (μg/kg)	0	0	0.13
TSH (mIU/L)	0.034	0.47	0.11
Total T3 (ng/mL)	1.02	0.75	0.94
Free T3 (pg/mL)	3	**2.2**	3.6
Total T4 (μg/dL)	9.8	**13.2**	10.9
Free T4 (ng/dL)	1.43	1.29	1.45
FT3/FT4 ratio	2.1	1.7	2.5
**Patient 2**	**Baseline**	**After selpercatinib start**	
Timepoint		1.5 months	3.5 months
Daily dose of LT4 (μg/kg)	1.47	1.47	1.34
Daily dose of LT3 (μg/kg)	0	0	0.11
TSH (mIU/L)	1.2	**4.6**	0.87
Total T3 (ng/mL)	1.15	0.86	0.73
Free T3 (pg/mL)	3.9	2.4	3.3
Total T4 (μg/dL)	10.4	9.5	**13.8**
Free T4 (ng/dL)	1.3	1.16	1.26
FT3/FT4 ratio	3.0	2.1	2.6
**Patient 3**	**Baseline**	**After selpercatinib start**	
Timepoint		1.6 months	3.7 months
Daily dose of LT4 (μg/kg)	1.84	1.84	1.58
Daily dose of LT3 (μg/kg)	0	0	0.10
TSH (mIU/L)	0.21	1.84	0.49
Free T3 (pg/mL)	3.9	2.6	3.2
Free T4 (ng/dL)	1.39	1.6	1.38
FT3/FT4 ratio	2.8	1.6	2.3

## Discussion

During the first year of treatment with TKIs, an increase in serum TSH level above the upper limit of the normal range occurred in half of the 45 LT4-treated patients enrolled in this study, and a slight but significant increase in TSH level was observed in the other patients. The mechanisms by which these alterations occur are partially unknown but probably reflect a class adverse event of all TKIs. We previously showed that angiogenesis inhibitors are able to reduce D2 expression and activity (Porcelli *et al.* 2023), and the same effect has also been demonstrated with selective RET inhibitors (Boucai *et al.* 2022). D2 is central to the production of T3 in thyroidectomised patients on LT4 therapy, and the fact that in approximately half our patients the increase of LT4 daily dose was unable to restore the pre-TKI serum FT3 levels supports the hypothesis of a defect in the D2-dependent T4-to-T3 conversion. On the other hand, the reduction in FT4 levels that we observed during TKI treatment could depend on an increased inactivation of T4 into reverse T3 (rT3) mediated by D3, as suggested by increased rT3 levels found in LT4-treated patients treated with selpercatinib (Boucai *et al.* 2022). In this study, we did not measure serum rT3 values or D1–D3 activities; therefore we cannot quantify a possible contribution of the inner ring deiodination activity by D3 to the TKI-related hypothyroidism. Regardless of the aetiological mechanism, we showed that the TKI-induced alteration of the thyroid axis has the peculiar characteristic of associating with an increase in TSH with a greater reduction in FT3 than in FT4, resulting in a decreased FT3/FT4 ratio. This characteristic distinguishes it from other forms of hypothyroidism, where the FT3/FT4 ratio increases as a consequence of the increase in the D2 activity aimed at maintaining a stable serum T3 concentration (Lazarus *et al.* 1992, Abdalla & Bianco 2014).

Increasing the dose of LT4 was effective in restoring both TSH and FT4 to pre-TKI levels in all patients. This indicates that additional doses of LT4 are able to overcome the TKI-induced mechanism underlying the reduction of FT4. However, increasing LT4 doses did not restore FT3 level and FT3/FT4 ratio to baseline levels in 55% of patients. The FT3/FT4 ratio – especially in thyroidectomised patients treated with LT4 – reliably reflects the thyroid status and is the best indicator of the amount of T4 that is converted to T3 by peripheral D2 deiodination (Jonklaas *et al.* 2021). Thus, the return of TSH to its pre-treatment levels does not necessarily correspond to rebalancing the thyroid axis. TSH is effectively inhibited by increasing doses of LT4. However, while the hypothalamic D2 is relatively insensitive to the T4-induced degradation, the peripheral D2 is highly sensitive to T4, which activates its degradation. As a consequence, the excess of T4 needed to suppress TSH secretion may reduce peripheral D2, thus the proportion of tissue conversion of T4 into T3 (Werneck de Castro *et al.* 2015). This may occur and further potentiate the inhibitory effect of the TKI on T3 production.

For this reasoning, in three consecutive patients treated with selpercatinib who showed a treatment-dependent reduction in total T3 and FT3 levels associated with increased TSH levels, we chose to reduce the dose of LT4 and to administer LT3 at an LT4/LT3 ratio of 11:1, lower than what is recommended in the context of hypothyroidism replacement therapy (i.e. 15:1) (Wiersinga *et al.* 2012), based on the hypothesis of an altered function of D2 caused by the TKI. This strategy normalised TSH while maintaining an FT3/FT4 ratio similar to baseline thanks to the increase in FT3 levels. In addition, the presence of normal/elevated total serum T4 level even after the reduction of LT4 dose suggests a serum T4 accumulation – probably due to a marked selpercatinib-dependent block in the T4-to-T3 or rT3 conversion. The small number of patients under study does not allow statistical significance to be reached, but these data provide a clear indication of the ability of LT4 + LT3 combination therapy to normalise the FT3/FT4 ratio while maintaining serum TSH levels at target.

We do not know what clinical benefit may derive from maintaining the FT3/FT4 ratio unchanged in patients submitted to TKI treatment. However, it is conceivable that any mechanism that reduces circulating T3 and inhibits D2 expression and/or activity may reduce the thyroid hormone action in thyroid hormone-target tissues. D2-expressing tissues are able to compensate for a small reduction in circulating T3 levels (Castagna *et al.* 2017), but this compensatory mechanism may be lost/reduced by an impaired D2 activity, as following TKI-induced D2 inhibition. Also, some thyroid hormone-target tissues such as liver and kidney do not express D2 and thus depend on circulating T3 (Salvatore *et al.* 2022).

Quantifying the contribution of a potential tissue hypothyroidism to the TKI-related adverse events is difficult. Many TKI-related adverse events overlap with symptoms of hypothyroidism, since they affect numerous target tissues of thyroid hormones. While for several tissues the mechanism of toxicity and its medical treatment have been at least partially clarified (Chen *et al.* 2016, Secombe *et al.* 2020), symptoms attributable to toxicity within the central nervous system such as fatigue, cognitive impairment and reduction of well-being still lack effective management (Giani *et al.* 2021). The thyroid hormone plays a key role in maintaining adult brain synaptic transmission (Walker *et al.* 1979), and more than two-thirds of the brain T3 in mice results from T4-into-T3 D2-dependent 5′-deiodination (Salas-Lucia *et al.* 2023). We previously demonstrated that TKIs inhibit D2 expression and reduce its activity (Porcelli *et al.* 2023). Here, we showed that patients that initiated a treatment with TKIs had lower FT3 levels than before TKI initiation, despite optimised LT4 treatment. In this context, we might speculate that central nervous system toxicity – as other TKI-related toxicities – may potentially be linked to an alteration of thyroid hormone serum/tissue concentrations. However, whether changes in circulating/tissue thyroid hormone concentrations in TKI-treated patients are responsible for or exacerbate some TKI-related adverse events remains to be demonstrated in prospective studies.

The retrospective nature of the study and the relatively small sample size limit the strength of its conclusions. Selection bias is reduced by having enrolled all patients who have been referred consecutively to our Institute, excluding only those who have not undergone blood tests in our laboratory. In addition, the sharp variations in thyroid hormone values recorded in these patients allowed statistical significance to be achieved despite the small sample size.

In conclusion, at least in thyroidectomised patients, TKIs induce a specific form of hypothyroidism characterised by an increase in TSH and a reduction in FT3 and FT3/FT4 ratio. Hypothyroid patients treated with LT4 monotherapy are probably more exposed to a potential interference on D2 activity than euthyroid subjects, since they depend on D2 for the conversion of LT4 into the active form T3. LT4 therapy effectively normalises TSH and FT4 values but restores FT3 values to pre-TKI therapy levels in only half of patients who have an increase in TSH level after TKI initiation. In our preliminary results, LT4 + LT3 combination treatment normalised the FT3 levels and the FT3/FT4 ratio to the values before the TKI initiation. Prospective trials are needed to answer the question of the clinical impact of compensating for the serum T3 decrease observed in patients treated with TKI.

## Supplementary materials



## Declaration of interest

The authors declare that there is no conflict of interest that could be perceived as prejudicing the impartiality of the work reported.

## Funding

The study was supported by the Federico II University Research Funding Programme 2022 to TP and by AIRC Individual Grant 2022 (Project no. 27729) to DS.

## Author contribution statement

TP, CL, AC, CDL and DS managed the patients. MADS performed laboratory analysis. TP and DS designed the study. TP and CL collected the data. TP analysed the data and wrote the manuscript. TP and DS revised the manuscript. All the authors approved the final manuscript and agreed to be accountable for the content of the work.
