# Supplementary material for: FT3 and FT3/FT4 ratio are decreased and not compensated by levothyroxine treatment in TKI-treated thyroid cancer patients
Source: Endocr Relat Cancer. 2025 May 14;32(6):e240323. doi: 10.1530/ERC-24-0323 (PMC12084765; doi:10.1530/ERC-24-0323)
Supplement: Supplementary file 1 [file supplementary_materials.pdf]

Supplementary Table 1. Coefficient of variations for the hormonal tests (Atellica IM analysers, Siemens Healthcare Diagnostics Inc, NY 10591, USA).

	Assay name	Reference intervals	Measuring intervals	Coefficient of variation
**TSH (mIU/L)**	TSH3-Ultra	0.55 - 4.78	0.01-150	≤8% for samples >0.3 mIU/L
**Total T3 (ng/mL)**	Total Triiodothyronine	0.61 - 1.81	0.1-8	N/A
**Free T3 (pg/mL)**	Free Triiodothyronine	2.3 - 4.2	0.2-20	≤12% for samples >2 pg/mL
**Total T4 (μg/dL)**	Total Thyroxine	4.5 - 10.9	0.3-30	N/A
**Free T4 (ng/dL)**	Free Thyroxine	0.89 - 1.76	0.1-12	<6% for samples >1 ng/dL
